# Improvement in pulmonary hypertension discrimination using multiple MRA pant-leg parameters of pulmonary artery

**DOI:** 10.1186/1532-429X-16-S1-O73

**Published:** 2014-01-16

**Authors:** Phillip Kilgas, Eric M Schrauben, Alejandro Roldán-Alzate, Naomi Chesler, Oliver Wieben, Christopher J Francois, Mark L Schiebler

**Affiliations:** 1 Radiology, UW-Madison School of Medicine and Public Health, Madison, Wisconsin, USA; 2 Medical Physics, UW-Madison, Madison, Wisconsin, USA; 3 Biomedical Engineering, UW-Madison, Madison, Wisconsin, USA

## Background

Pulmonary arterial hypertension (PAH) is a potentially severe disease that can lead to exercise intolerance, venous congestion, and heart failure. As a result of increased pulmonary pressures, dilation of the pulmonary arterial vasculature occurs. The extraction of these anatomical changes from magnetic resonance angiogram (MRA) may eliminate the need for invasive measurements. The aim of this study was to explore the use of volumetric imaging of the pulmonary trunk and proximal right and left arteries using MRA as a new metric for diagnosing PAH.

## Methods

**Figure 1:**
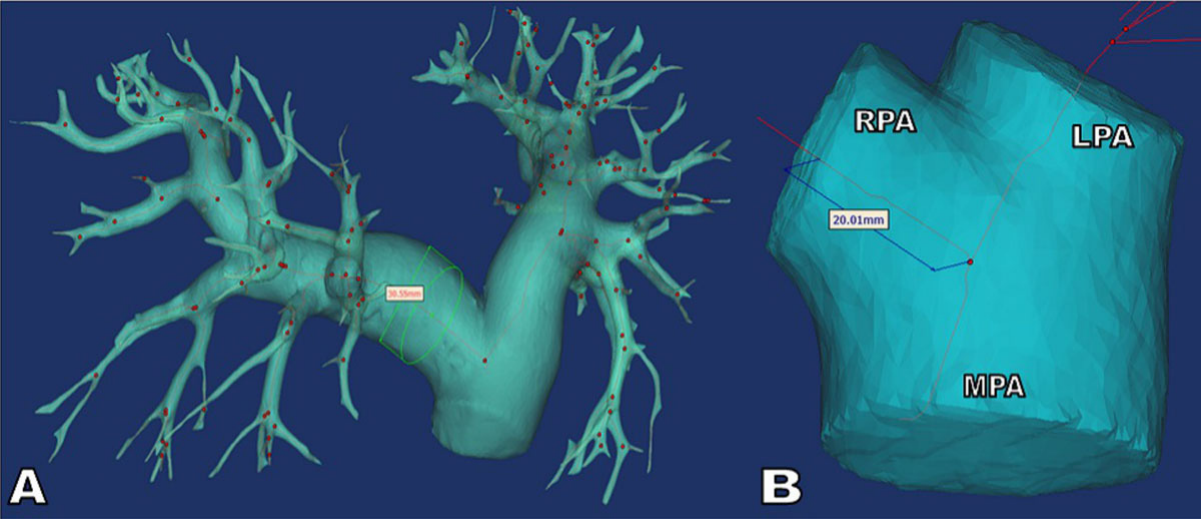
**A diameter (a) and distance from bifurcation (b) measurement on the right pulmonary artery of a PAH patient**. The diameter and distance measurements are based on a best fit centerline.

Following IRB approval, eight PAH patients referred for right heart catheterization (RHC) for systemic sclerosis were evaluated with 3D contrast-enhanced MRA. For comparison, eight healthy volunteers underwent the same MRA protocol. All MRA exams were performed on 3.0T clinical scanners (GE Healthcare, Waukesha, WI) following the administration of gadolinium-based contrast agent at 1.5 ml/sec (gadobenate dimeglumine, Bracco, Milan). Scan parameters of the SPGR sequence included TR/TE of 2.9/1.0 ms, average field of view = 34 × 27 cm, slice thickness = 2.0 mm, 140-160 slices, flip angle = 28°, and true spatial resolution of 1.3 × 1.8 × 2.0 mm^3^, which was interpolated to 0.7 × 0.7 × 1.0 mm^3 ^by zero-filling. Approximate breath-hold time was between 15-21 seconds for each scan. These exams were not cardiac gated. Post-processing was evaluated using dynamic magnitude images in a commercial software (Mimics, Materialise, Belgium) to calculate diameter (main, left, and right pulmonary artery: MPA, LPA, RPA), volume, surface area, branch sum, and area sum pant-leg (P-L) measurements (Figure 1a). Each P-L was obtained by centerline semi-automated measurements two centimeters in each direction of bifurcation (Figure 1b). These parameters were evaluated as mean ± standard deviation. Differences in these measurements were statistically analyzed using a paired Student's t-test.

## Results

Figure 1a demonstrates volumetric segmentation and quantification of the pulmonary arterial tree using thresholding and centerline extraction. Figure 1b shows a close-up image of P-L measurement from the pulmonary trunk bifurcation. Table 1 summarizes average (± standard deviation) measurements between PAH patients and healthy controls. In all eight PAH cases, dilation and volume increased compared to healthy volunteers. Each measurement proved to be statistically significant at the 5% level (p ≤ .05). Interestingly, the LPA measurement, at the cuff of the pant-leg, showed the best discriminatory power in this analysis.

**Table 1:** Summarized results

	PAH Patients	Normal Volunteers	P-Values
**MPA (mm)**	36.10 ± 3.66	30.15 ± 3.24	0.003951
**LPA (mm)**	27.45 ± 2.83	19.08 ± 2.81	0.000036
**RPA (mm)**	27.27 ± 2.74	19.73 ± 2.86	0.000097
**P-L Volume (mm^3^)**	44155.81 ± 12340.40	23978.23 ± 5009.86	0.001912
**P-L Surface Area (mm^2^)**	7336.98 ± 1260.08	5154.16 ± 764.20	0.001366
**P-L Branch Sum (mm)**	54.72 ± 5.31	38.81 ± 5.57	0.000042
**P-L Area Sum (mm^2^)**	1186.46 ± 234.13	602.61 ± 170.31	0.000055

## Conclusions

Semi-automated computer based measurements of the pulmonary arterial tree, using pant-leg pulmonary arterial volume, resulted in an improved ability to discriminate for the presence of PAH when compared with the current standard of pulmonary artery diameter.

## Funding

NIH: HL105598, HL115061.

